# The relationship between frailty and social participation: focus on subjective health

**DOI:** 10.1186/s13104-023-06407-x

**Published:** 2023-06-26

**Authors:** Yuho Shimizu, Masashi Suzuki, Yukako Hata, Toshiro Sakaki

**Affiliations:** 1 Graduate School of Humanities and Sociology, The University of Tokyo, 7-3-1 Hongo, Bunkyo-ku, Tokyo, 113-0033 Japan; 2 Japan Society for the Promotion of Science, 5-3-1 Kojimachi, Chiyoda-ku, Tokyo, 102-0083 Japan; 3 Healthcare Business Development Department, Manager, Sompo Holdings, Inc, 1-26-1 Nishi-Shinjyuku, Shinjyuku-ku, Tokyo, 160-8338 Japan; 4 SAT laboratory LLC, 3-20 Matsunouchi-cho, Ashiya, 659-0094 Hyogo Japan

**Keywords:** Older adults, Frailty, Social participation, Subjective health, Japan

## Abstract

**Objective:**

Active participation of the older adults in the society is crucial; however, frailty prevents social participation. Meanwhile, many older adults participate daily in social activities, even with frailty. This study aims to examine whether older adults with frailty have lower social participation than those without frailty in Japan. We also investigated whether older adults with frailty and higher subjective health participate in society to the same extent as the general older population. This study included 1,082 Japanese individuals aged 65 years and older participating in the online survey. Participants answered questions on social participation, frailty, subjective health, and demographics.

**Results:**

Participants in the robust group had higher social participation rates than those in the frailty and pre-frailty groups. Meanwhile, frail older participants with higher subjective health had similar social participation as the robust participants. Many older adults acquire frailty despite their individual effort. Meanwhile, improving subjective health may be effective, even with frailty. The relationship between subjective health, frailty, and social participation is primitive and further studies are needed.

**Supplementary Information:**

The online version contains supplementary material available at 10.1186/s13104-023-06407-x.

## Introduction

The world population is rapidly aging, especially in Japan, where 28.9% of the total population in 2021 was aged 65 or older [1]. The current society deems the active participation of older adults as important and meaningful. Social participation refers to people performing activities in their daily lives and taking on social roles [2], which contributes to the promotion of well-being in older adults [3] and reduction of depressive tendencies [4]. The social participation of older adults is crucial to compensate for the decline in the working-age population, which is serious in many developed countries including Japan. Thus, we should investigate how to promote social participation of older adults.

Frailty was our primary focus, as it prevents their social participation (increased vulnerability to health problems due to various age-related functional changes) [5]. Since social participation requires a certain degree of cognitive function and physical activity [6, 7], older adults with frailty are likely to have lower social participation. Indeed, previous studies of older adults in China and Taiwan suggested higher social participation with higher cognitive and physical function [3, 8]. In this study, we evaluate the finding that older adults with frailty have lower social participation is replicated in Japan.

Meanwhile, many older adults participate in social activities on a daily basis even with frailty. The difference between these frail adults and other older adults is a striking question. As one of the characteristics of such older adults, we focused on subjective health (comprehensive and self-rated health measures defined by a wide range of factors, including physical and mental health, income, and living environment) [9]. The subjective health of older adults has received increasing attention in the academic field of public health [10]. Indeed, older adults with higher subjective health have higher social participation [11, 12], as well as higher quality of life [13] and life satisfaction [14]. It should be noted that while subjective health and objective health-related indicators generally have a positive correlation [15], it is reported that frail older adults with low depressive tendencies maintain the same level of subjective health as the general older population [16]. Therefore, we exploratively examine the possibility of frail older adults having higher subjective health and participating in the society similarly as the general older population.

We hereby evaluate the following hypotheses by conducting an online survey for older adults aged 65 years and more. Specifically, we examine whether older adults with frailty have lower social participation than robust older adults in Japan (Hypothesis 1) and whether older adults with frailty having higher subjective health participate in society similarly as the general older population (Hypothesis 2).

## Main text

### Methods

**Participants.** This study included 1,082 Japanese aged 65 years and older (548 males and 534 females). Participants were registered respondents of Intage Inc., a major Japanese research company. This survey was conducted online in June 2022. Older adults certified as requiring long-term care (*N* = 18) were excluded from the study. Finally, 1,064 participants were included in the study (542 males and 522 females), with a mean age of 70.59 years (*SD* = 4.68). Participants’ demographic information and the results of the study related to the certification of requiring long-term care are presented in the Open Science Framework (OSF) repository (https://osf.io/xycsk/). Ethical approval was obtained from the authors’ institution and all methods were performed in accordance with the relevant guidelines and regulations. Informed consent was obtained from all participants in the form of checking a box in the online survey.

A power analysis was conducted for conducting a one-factor analysis of variance (*k*_group_ = 3 (frailty, pre-frailty, robust), *f* = 0.25 (medium effect [17]), α = 0.05, *β* = 0.80). Thus, the required sample size in each group was 53. The sample size of each group in this study met this criterion (for the exact sample size in each group, see below). The survey was developed for this study and all items included in the survey were posted on the OSF.

**Measurements and procedure.** Participants completed an online survey and responded to each of the following items. Social participation was measured using 14 items (five-point Likert scale) [18]. The mean was calculated (α = 0.92), with higher scores indicating higher social participation. The specific content of social participation (e.g., volunteer groups, sports groups) was also measured, and the results were posted on the OSF. Frailty was measured using five items (two-point Likert scale) of the Frailty Screening Index [19]. The total is the frailty score, with higher scores indicating a higher degree of frailty. Scores ≥ 3 were considered to indicate frailty (*N* = 164); 1–2, pre-frailty (*N* = 675); and 0, robust (*N* = 225). Subjective health was measured using five items (four-point Likert scale) of the S-WHO-5-J [20]. The mean was calculated (α = 0.86), with higher scores indicating higher subjective health. Demographic items, including participants’ subjective wealth, cohabitation, work status, age, and gender, were evaluated. Histograms of each indicator were posted on the OSF.

## Results

The summary statistics of each group are illustrated in Table 1. The results were similar when data from all the participants without screening were analyzed (for details, see OSF). When comparing social participation among the three groups (frailty, pre-frailty, and robust); controlling the participants’ subjective health, subjective wealth, cohabitation, work status, age, and gender, the main effect of group was significant (*F*(2, 1055) = 46.19, *p* < .001, *η*^2^ = 0.09). Multiple comparisons using Tukey’s test revealed that participants in the robust group had higher social participation than those in the frailty (*t*(1055) = 3.58, *p* = .001, *d* = 0.83) and pre-frailty groups (*t*(1055) = 2.79, *p* = .01, *d* = 0.35), supporting Hypothesis 1. The same results were obtained without considering control variables (see OSF).

**Table 1:** Summary statistics by each group

Robust (*N* = 225)		*M*	*SD*	1	2
1	social participation	3.21	0.64	―	
2	subjective health	2.84	0.46	0.55^**^	―
3	age	70.44	4.31	0.08	0.14^*^
Pre-frailty (*N* = 675)		*M*	*SD*	1	2
1	social participation	2.95	0.75	―	
2	subjective health	2.68	0.56	0.53^**^	―
3	age	70.61	4.76	0.17^**^	0.11^*^
Frailty (*N* = 164)		*M*	*SD*	1	2
1	social participation	2.62	0.79	―	
2	subjective health	2.38	0.54	0.56^**^	―
3	age	70.73	4.83	0.17^*^	0.07

*Note*. *p < .05, **p < .01

To investigate the interaction between subjective health and frailty score, a multiple regression analysis was performed with social participation as the dependent variable and subjective health, frailty score, the interaction between them, subjective wealth, cohabitation, work status, age, and gender as the independent variables (Table 2). As a result, the interaction between subjective health and frailty score on social participation was not significant (*β* = 0.01, 95%CI = [-0.04, 0.06], *p* = .70). Meanwhile, frail participants who had higher subjective health had higher social participation scores than many robust participants. Specifically, the estimated social participation score of participants with higher subjective health (+ 1*SD*; 2.92) in the frailty group was 3.06, and 87 (38.67%) robust participants scored below this value. The estimated social participation score of the participants with higher subjective health (+ 1*SD*; 3.24) in the pre-frailty group was 3.35, and 131 (58.22%) robust participants were below this value (for details, see OSF). In summary, Hypothesis 2 is supported by the fact that older adults with frailty and higher subjective health participate in society to the same extent as robust older adults. In other words, even if a person is frailty (or pre-frailty), if his/her subjective health is high, he/she can participate in society as well as robust people.

**Table 2:** Multiple regression analysis results on social participation

		*β*	95%CI	VIF
1	subjective health	0.47**	[0.42, 0.53]	1.21
2	frailty score	-0.09**	[-0.15, -0.04]	1.14
3	interaction (1 × 2)	0.01	[-0.04, 0.06]	1.06
4	subjective wealth	0.14**	[0.09, 0.19]	1.16
5	cohabitation	-0.03	[-0.08, 0.02]	1.03
6	work status	0.13**	[0.08, 0.18]	1.10
7	age	0.12**	[0.07, 0.17]	1.08
8	gender	-0.10**	[-0.15, -0.05]	1.08
	adjusted *R*^*2*^	0.37**	[0.33, 0.42]	-

*Note*. **p < .01

## Discussion

We focused on frailty as a factor that influences older adults’ social participation. We conducted an online survey with Japanese older adults and found that frail older adults have lower social participation than robust older adults. Meanwhile, we showed that older adults with frailty and higher subjective health participate in social activities to the same extent as the general older population. The results suggest that even older people with low physical health may be able to participate well in society if their subjective health is high enough.

In previous studies [11, 12], older adults with higher subjective health had higher social participation. However, this association is only a correlation, and it is unclear whether enhancing the subjective health of older adults will increase the degree of social participation. Additionally, strategies to enhance their subjective health should be investigated. Some aspects of physical health will be maintained in each older adult while others will not, such as vision, hearing, and other physical functions. Therefore, one of the effective strategies to increase older adults’ subjective health would be having them focus on their healthier aspects. Academic researchers in public health should continue to examine the subjective health of older adults in detail.

## Limitations

Despite these findings, this study has three major limitations. First, the participants were limited to older adults who were able to participate in an online survey. In the future, to cover a broader sample, a mail survey should be conducted among older adults throughout Japan. Meanwhile, the number of participants in the pre-frailty group was 4.12 times that of the robust group; thus, the sample was not biased toward healthy participants. Rather, a higher percentage of participants was frailty or pre-frailty. This may be related to the fact that the survey was conducted during the COVID-19 epidemic. There may have been a temporary increase in the number of participants answering “yes” to the frailty questionnaire items on “Do you go for a walk for your health at least once a week?” and “In the last 2 weeks have you felt tired without a reason?” Therefore, we do not believe that our sample was biased.

Second, frailty was measured using the five items of the Frailty Screening Index [19]. This scale is frequently used with the Japanese older adults and is less burdensome for the participants. However, several measures of frailty have been developed [21] and the robustness of this study should be re-examined while taking a more multifaceted view of frailty.

Third, items on subjective health do not include ones regarding the comparison of the health between the self and other older adults around the participants [22]. A longitudinal study has shown that older adults who consider themselves healthier than other older adults have lower subsequent mortality rates [23]. Accordingly, we should also examine the effect of feeling healthier than other older citizens on social participation. The subjective health of older adults should be measured extensively and investigated in more detail.

We exploratively focused on subjective health as a characteristic of older adults participating in social activities even with frailty. It is crucial to prevent frailty in promoting social participation. However, the decline in physical function of older adults varies greatly with each person, and some older adults acquire frail at a relatively young age [19]. Based on this study, it may be effective to improve subjective health, even with frailty, by focusing on the positive aspects of one’s health status. The relationship between subjective health, frailty, and social participation is primitive, and further studies are needed.

## Electronic supplementary material

Below is the link to the electronic supplementary material.

Supplementary Material 1

## Data Availability

The data and materials underlying this article are available in the Open Science Framework (OSF) repository (https://osf.io/xycsk/).
