# Supplementary material for: The relationship between frailty and social participation: focus on subjective health
Source: BMC Res Notes. 2023 Jun 26;16:123. doi: 10.1186/s13104-023-06407-x (PMC10294298; doi:10.1186/s13104-023-06407-x)
Supplement: Supplementary file 1 — Supplementary Material 1 [file 13104_2023_6407_MOESM1_ESM.docx]

**Contents**

[Appendix A: Scales 2](#_Toc136423192)

[Appendix B: Participants’ Demographic Information 3](#_Toc136423193)

[Appendix C: Certification of Requiring Long-Term Care 4](#_Toc136423194)

[Appendix D: Contents of Social Participation 5](#_Toc136423195)

[Appendix E: Histograms (all participants) 8](#_Toc136423196)

[Appendix F: Summary Statistics (all participants) 10](#_Toc136423197)

[Appendix G: Comparison of Social Participation Between Groups (without consideration of control variables) 11](#_Toc136423198)

[Appendix H: Scatter Plots in the Robust Group Only (regarding Hypothesis 2) 12](#_Toc136423199)

[References 13](#_Toc136423200)

# Appendix A: Scales

**Frailty**

1. Have you lost 2 kg or more in the past 6 months?
2. Do you think you walk slower than before?
3. Do you go for a walk for your health at least once a week?
4. Can you recall what happened 5 minutes ago?
5. In the last 2 weeks have you felt tired without a reason?

**Social Participation**

1. I am satisfied with the company of my friends.
2. I sometimes feel that I have been helpful to some group, organization, or association.
3. I learn about my interests and concerns in my own way.
4. I sometimes feel more confident about my health in my own way.
5. I sometimes feel that I have helped the community in which I live.
6. I sometimes think that I could learn something that might be useful to me.
7. I have friends that I would enjoy doing some activities with.
8. I sometimes think I have gained confidence in my own physical strength.
9. I sometimes think I could have improved my education.
10. In my own way, I help people in need.
11. I have a good time with my friends.
12. In my own way, I am doing something useful for society.
13. Sometimes I think it has helped me maintain my health.
14. Sometimes I think I have satisfied my intellectual curiosity.

**Subjective Health**

1. I have felt cheerful and in good spirits.
2. I have felt calm and relaxed.
3. I have felt active and vigorous.
4. I woke up feeling fresh and rested.
5. My daily life has been filled with things that interest me.

**Subjective Wealth**

1. How do you feel about your current financial situation?

**Cohabitation**

1. Do you currently live alone?

**Work Status**

1. Do you currently work in any capacity?

# Appendix B: Participants’ Demographic Information

Demographic information for the participants is shown in Table S1.

Table S1

Demographic information for the participants

# Appendix C: Certification of Requiring Long-Term Care

In the main manuscript, participants with the certification of requiring long-term care (*N* = 18) were excluded from the analysis. We compared social participation between participants with the certification of requiring long-term care (*N* = 18, *M* = 2.58, *SD* = 0.85) and the other participants (*N* = 1064, *M* = 2.95, *SD* = 0.75), controlling for participants’ subjective health, subjective wealth, cohabitation, work status, age, and gender. As a result, participants with the certification of requiring long-term care had lower social participation than the other (*F*(1, 1074) = 6.76, *p* = .001, *η*^2^ = .006).

We compared subjective health between participants with the certification of requiring long-term care (*N* = 18, *M* = 2.29, *SD* = 0.50) and the other participants (*N* = 1064, *M* = 2.67, *SD* = 0.55), controlling for participants’ subjective wealth, cohabitation, work status, age, and gender. As a result, participants with the certification of requiring long-term care had lower subjective health than the other (*F*(1, 1075) = 9.36, *p* = .002, *η*^2^ = .009). However, these effect sizes were small, and thus more detailed studies are needed to determine the differences in social participation and subjective health between participants with the certification of requiring long-term care and the other participants. Due to the small sample size of participants with the certification of requiring long-term care, a detailed analysis should be conducted in future studies.

# Appendix D: Contents of Social Participation

Along with the previous studies [1,2], participants were asked to respond to the following eight items regarding their specific social participation (1. Volunteer groups, 2. Sports groups, 3. Hobby groups, 4. Senior citizen clubs, 5. Neighborhood associations, 6. Study/cultural groups, 7. Health promotion groups, 8. Teaching skills/passing on experiences to others). Participants responded using the four-point Likert-scale (1. Never, 2. Several times/year, 3. 1-3 times/month, 4. Once a week or more). Results for these items are shown in Table S2.

Table S2

Contents of social participation

The mean of the eight items was calculated as the frequency score of social participation (min = 1, max = 4). The frequency scores were *M* = 1.34 (*SD* = 0.36) in the robust group, *M* = 1.26 (*SD* = 0.35) in the pre-frailty group, and *M* = 1.20 (*SD* = 0.28) in the frailty group. In the following, we conducted the same analysis as in the main manuscript with frequency scores as the dependent variable.

We compared social participation (frequency score) between three groups (robust, pre-frailty, and frailty), controlling for participants’ subjective health, subjective wealth, cohabitation, work status, age, and gender. As a result, the main effect of group was significant (*F*(2, 1055) = 8.33, *p* < .001, *η*^2^ = .02). Multiple comparisons were conducted and there were no significant differences in frequency scores between any of the groups (all *p*s > .16). Meanwhile, we also compared social participation (frequency score) between three groups without considering control variables. Results showed that the main effect of group was significant (*F*(2, 1061) = 7.57, *p* < .001, *η*^2^ = .01). Multiple comparisons were conducted and the robust group had higher frequency score than the frailty (*t*(1061) = 3.83, *p* < .001) and pre-frailty groups (*t*(1061) = 2.74, *p* = .006). Also, the pre-frailty group had higher frequency score than the frailty group (*t*(1061) = 2.08, *p* = .04). Thus, we can say that Hypothesis 1 is generally supported even when the frequency score is used as the dependent variable.

The relationship between participants’ subjective health and social participation (frequency score) is shown in Figure S1. From the figure, we can say that participants in the frailty group but high subjective health have higher frequency scores than a lot of participants in the robust group. Specifically, the estimated value of frequency score based on the regression line for the frailty participants with high subjective health (+1*SD*; 2.92) was 1.30, and 125 (55.56%) of the robust participants had a score below this value. Also, the estimated value of frequency score based on the regression line for the pre-frailty participants with high subjective health (+1*SD*; 3.24) was 1.34, and 125 (55.56%) of the robust participants had a score below this value. These results indicate that participants with frailty but high subjective health participate in social activities as frequently as the robust participants, thus supporting Hypothesis 2.


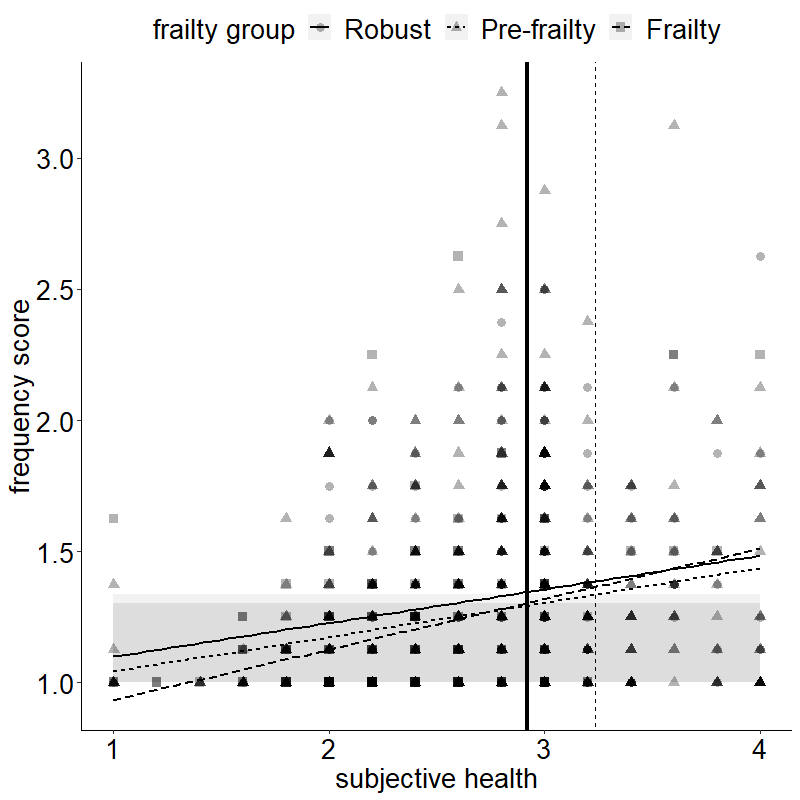


Figure S1. Scatter plots on subjective health and social participation (frequency score). The lines parallel to the y-axis are x = 2.92 (bold) and x = 3.24 (dotted). The former is +1*SD* of subjective health in the frailty group and the latter is +1*SD* of subjective health in the pre-frailty group.

# Appendix E: Histograms (all participants)

Histograms of all participants on each indicator are shown below.


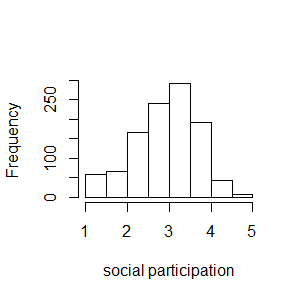


Figure S2. Histogram on social participation.


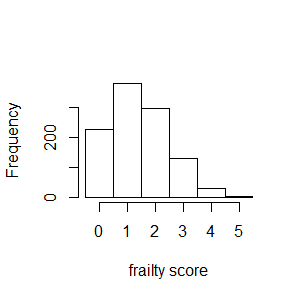


Figure S3. Histogram on frailty score.


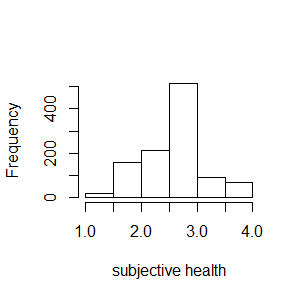


Figure S4. Histogram on subjective health.


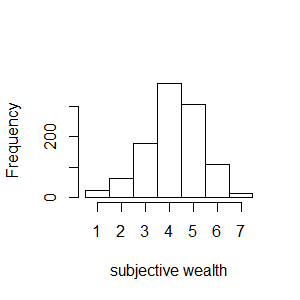


Figure S5. Histogram on subjective wealth.


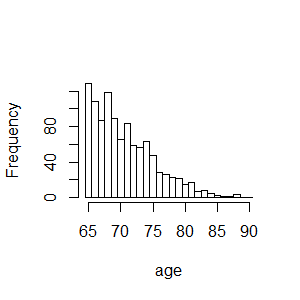


Figure S6. Histogram on participants’ age.

# Appendix F: Summary Statistics (all participants)

Summary statistics for all participants are shown in Table S3.

Table S3

Summary statistics for all participants (*N* = 1064)

*Note*. **p* < .05, ***p* < .01.

# Appendix G: Comparison of Social Participation Between Groups (without consideration of control variables)

An analysis of variance was performed with group (robust, pre-frailty, and frailty) as the independent variable and social participation as the dependent variable. In this case, control variables were not considered and the means were simply compared. Results showed that the main effect of the group was significant (*F*(2, 1061) = 30.57, *p* < .001, *η*^2^ = .05). Multiple comparisons were conducted and the robust group had higher social participation than the frailty (*t*(1061) = 7.82, *p* < .001) and pre-frailty groups (*t*(1061) = 4.48, *p* < .001). Also, the pre-frailty group had higher social participation than the frailty group (*t*(1061) = 5.26, *p* < .001). Based on the above, as in the main manuscript, Hypothesis 1 is supported.

# Appendix H: Scatter Plots in the Robust Group Only (regarding Hypothesis 2)

Regarding Hypothesis 2, it can be said that frail participants who have higher subjective health have higher social participation scores than many robust participants (Figure S7).


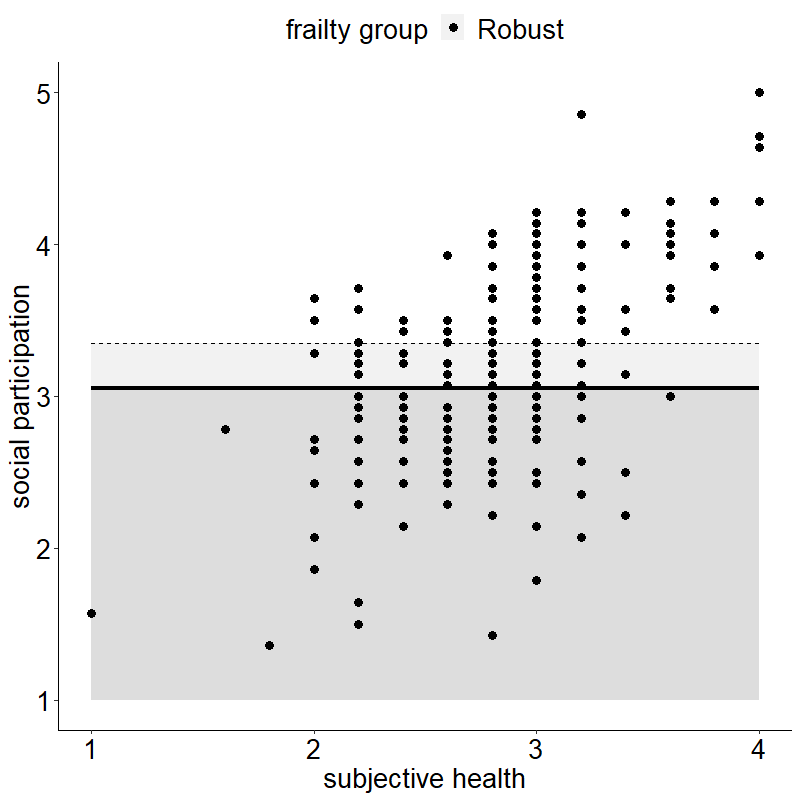


Figure S7. Scatter plots on subjective health and social participation in the robust group only. The lines parallel to the x-axis are y = 3.06 (bold) and y = 3.35. The former is the estimated social participation score based on the participants with higher subjective health (+1*SD*; 2.92) in the frailty group. The latter is the estimated social participation score based on the participants with higher subjective health (+1*SD*; 3.24) in the pre-frailty group.

# References

1. Takesue, A., Hiratsuka, Y., Inoue, A., Kondo, K., Murakami, A., & Aida, J. (2021). Is social participation associated with good self-rated health among visually impaired older adults? The JAGES cross-sectional study. *BMC Geriatrics*, *21*(1), 1–9. <https://doi.org/10.1186/s12877-021-02554-7>
2. Yoshida, Y., Hiratsuka, Y., Kawachi, I., Murakami, A., Kondo, K., & Aida, J. (2020). Association between visual status and social participation in older Japanese: The JAGES cross-sectional study. *Social Science and Medicine*, *253*, 112959. <https://doi.org/10.1016/j.socscimed.2020.112959>
